# A comparative study of the physical activity guidelines for children and adolescents from five countries and WHO

**DOI:** 10.3389/fpubh.2024.1421843

**Published:** 2024-07-12

**Authors:** Donglin Hu, Shi Zhou, Zachary J. Crowley-McHattan, Zhiyun Liu

**Affiliations:** ^1^Department of Physical Education, Nanjing Agricultural University, Nanjing, China; ^2^Faculty of Health, Southern Cross University, Lismore, NSW, Australia; ^3^School of Physical Education and Educational Science, Tianjin University of Sport, Tianjin, China

**Keywords:** physical activity, adolescents, physical activity guidelines, children, physical activity participation

## Abstract

This study presented a narrative review of the six latest sets of guidelines on physical activity for children and adolescents from Australia, Canada, China, the United Kingdom, the United States and WHO, and analysed the history of the development of the guidelines; the policy context in which the guidelines were developed; and the main subjects of the guidelines. The core elements of the latest guidelines were identified and compared, including frequency, intensity, duration (time), and type of physical activity, assessment intensity of physical activity, and risk prevention for physical activity participation. There is an urgent need for obtaining the latest scientific evidence to support and update the contents of the Chinese guidelines. We therefore make the following recommendations for future revisions of the Chinese guidelines based on the findings of this study: (1) to update the recommended frequency, intensity, duration, and type of physical activities that meet the needs for Chinese children and adolescents; (2) to update the methods for assessing physical activity intensity, and identifying and managing the risk factors in participating in physical activity; and (3) to incorporate analyses and reviews of school physical education curriculum for effectively achieving the adequate levels of physical activity.

## 1 Introduction

Physical activity was defined by Caspersen and colleagues in 1985 as ‘any energy-consuming form of bodily movement resulting from the activity of the skeleton’ (1). The benefits of participating in adequate levels of daily physical activity have been repeatedly demonstrated in the literature (2, 3). The composition of physical activity, from a biological viewpoint, can be described with four core elements: frequency, intensity, duration (time) and type, a principle that has been used by the World Health Organization (WHO) and scholars worldwide to date (4). On this basis, according to the WHO, nearly three-quarters of adolescents (aged 11 to 17 years) worldwide do not meet the WHO’s physical activity guidelines and recommendations (4, 5). Physical inactivity is influenced by socioeconomic development, changes in transportation modes, technological innovations, and urbanisation (4). Numerous studies have shown that physical inactivity in children and adolescents contributes to many health disorders, such as dyslipidaemia, obesity, cardiovascular disease, poor mental health and inadequate cognitive development (3, 6, 7). The continued decline in physical activity levels among children and adolescents has caused concerns in many countries. Especially in the developed Western countries, corresponding physical activity guidelines have been developed in recent years to promote physical health of children and adolescents.

In 2008, the United States Department of Health and Human Services (HHS) introduced the first set of *Physical Activity Guidelines for Americans*, establishing a scientific and standardised framework for physical activity recommendations. This landmark publication provided guidance for Americans and became a reference for many other countries and organisations (8, 9). Since its release, the guideline has been supported by various health promotion policies from the US federal government, including the *2016 National Physical Activity Plan* from the National Physical Activity Plan Alliance, the *Active People, Healthy Nation Campaign* from the Centres for Disease Control and Prevention (CDC), and the *Healthy People 2020* policy from HHS, which set objectives for increasing physical activity in Americans over a decade from 2010. In 2018, HHS updated the guidelines to the 2nd edition based on the latest scientific evidence. The new guidelines emphasise that regular physical activity over months and years can produce long-term health benefits.

Since 2010, Australia, Canada, the United Kingdom, WHO, and some other countries have developed guidelines for physical activity for children and adolescents. In 2010, WHO published the *Global Recommendations on Physical Activity for Health*, which was the first WHO guidelines for all age groups, including children and adolescents (2). In 2018, WHO put forward the *2018–2030 Global Action Plan on Physical Activity* (5), which called for a 15% reduction in physical inactivity among adults and adolescents worldwide by 2030. In this context, the WHO updated the guidelines in the *WHO 2020 Guidelines on Physical Activity and Sedentary Behaviour*, which provide evidence-based recommendations for promoting healthy physical activity levels in different age groups.

In 2016, Canada completed an update of its physical activity guidelines in the context of a broader national policy to promote physical activity and health (10). Particularly in respect of children and adolescents, the *Canadian 24-Hour Movement Guidelines for Children and Youth* integrate several policy documents, including *The Canadian Sport Policy*, *Canadian Sport for Life, ParticipACTION’s Public Awareness Campaign*, and *The Canadian Assessment of Physical Literacy*. These guidelines provide recommendations for achieving physical fitness in Canadian children and adolescents (11, 12).

In 2016, the *Canadian 24-Hour Movement Guidelines for Children and Youth* were the impetus for developing similar guidelines in Australia (13, 14). Australia drew on the Canadian guide’s core recommendations to encourage their children and youth to be physically active, develop lifelong physical habits, and meet the National Physical Literacy Standards (14). In 2019, the Australian Government Department of Health developed The *Australian 24-h Movement Guidelines for Children and Young People (5–17 years): An integration of physical activity, sedentary behaviour, and sleep* (15).

To promote a healthy population and healthy lifestyle, the UK government has introduced several policies related to weight control and healthy living since 2010, such as *Change4Life, PE & Sport Strategy for Young People (PESSYP)*, *Sporting Future: A New Strategy for an Active Nation* (16, 17). In 2011, the Department of Health and Social Care integrated these policies and published the first physical activity guidelines. In 2019, the UK government collaborated with research institutions in England, Scotland, Wales, and Northern Ireland to release the latest version of the *UK Chief Medical Officers’ Physical Activity Guidelines*, which provide advice and assistance for physical activity in children and adolescents (18).

The physical activity and physical health of children and adolescents in China have long been of broad concern to the society (3, 8). In 2016, China issued the *National Fitness Plan (2016–2020)* (19), which identified adolescents as a key population group and called for vigorous promotion and popularisation of physical activity to enhance their physical health. In the same year, another important policy document, the *‘Healthy China 2030’ plan* (20), highlighted the importance of physical activity for children and adolescents. In 2018, in the context of national strategies such as *National Fitness* and *Healthy China*, the Working Group on the Development of Physical Activity Guidelines synthesised the research results of guidelines from Western developed countries up to 2016, and released China’s first *Physical Activity Guidelines for Children and Adolescents* (21).

*The Physical Activity Guidelines for Children and Adolescents* (21) were developed using a rigorous and evidence-based process, which provided authoritative recommendations on the frequency, intensity, time, and type of physical activity necessary to promote health and mitigate health risks in this population. The development of physical activity guidelines was based on the best available evidence at the time, with the potential for updates as new evidence emerges. However, due to limited research on physical activity among Chinese children and adolescents, the 2018 Physical Activity Guidelines for Chinese children and adolescents heavily relied on guidelines and systematic reviews from other countries before 2016, such as those from the WHO, United Kingdom, United States, Australia, Canada. With the emergence of new evidence after 2016, these countries and organisations have subsequently updated their guidelines.

Therefore, in this study, we targeted these five countries and organisations as the main source of reference for the latest guidelines for comparison with current Chinese guidelines (21), aiming to identify the similarities and gaps. By doing so, this study aimed to provide valuable insights that could inform and improve the future revision of the Chinese guidelines, ensuring they were up-to-date and reflecting current scientific knowledge and best practices for promoting physical activity in children and adolescents.

## 2 Methods

### 2.1 Data collection and analysis

In our research, we undertook a narrative review of physical activity guidelines for children and adolescents from Australia, Canada, the United Kingdom, the United States, the WHO, and China. This selection was guided by their demonstrated leadership and diversity in public health, vital for an exhaustive comparative analysis. The inclusion of China was particularly crucial, as our research focuses on Chinese physical activity guidelines. Our comparison of China’s guidelines with those of other countries and the WHO was intended to pinpoint areas for potential improvement in the Chinese context.

Our literature review focused on the materials published in English and Chinese languages, with consideration of the accessibility of the literature, and the language proficiency of the research team. The review was based on the following key steps:

**Data Retrieval**: Initially, a thorough search was conducted on government health department websites and official websites of these countries and organisations. The search aimed to collect literature on physical activity guidelines, using keywords such as “physical activity guidelines,” “children and adolescents” and “health.”

**Screening Criteria**: Selection was based on publication dates and specific content related to physical activity guidelines. Special attention was paid to guidelines published in the last five.

years to ensure the relevance and timeliness of the data.

**In-depth Analysis**: Detailed analysis of the selected guidelines was undertaken, comparing recommended types, frequencies, durations, and intensities of physical activities. This step involved a careful reading of the text and a comparative analysis of the recommended standards.

**Quality Control**: To ensure reliability and validity of the findings, multiple quality control measures were applied during data collection and analysis. This included double data entry and cross-verification to minimise biases in collection and interpretation.

**Literature Synthesis**: By comparing different guidelines, the study synthesised the variations and similarities among them, especially reflecting the socio-cultural and educational contexts they represent.

**Reflection and Critical Evaluation**: Based on the synthesis, the study involved critical reflection and evaluation of the effectiveness and applicability of these guidelines, providing suggestions for improvements to China’s physical activity guidelines.

Through these meticulous methodological steps, the study aimed to offer a comprehensive and in-depth perspective for understanding and evaluating the core elements and characteristics of physical activity guidelines across different countries, thereby providing a theoretical basis and practical guidance for optimising China’s physical activity guidelines.

## 3 Results and discussion

### 3.1 Guideline developers

As can be seen in Table 1, the developers of the guidelines from Australia, Canada, China, the United States, the United Kingdom, and WHO (Table 1) included both national government departments and professional organisations such as scientific research institutes, sports and health industry associations, and higher education institutions. These guidelines were developed by national health authorities, whose main objective being to promote physical fitness and health for children and adolescents in their countries. The non-governmental organisations (NGOs) regularly hold public meetings to systematically review the latest scientific literature on physical activity and health and contribute significantly to the guidelines’ design, implementation, and evaluation (4, 5). The involvement of government departments and NGOs in developing these guidelines is crucial for improving the physical health of children and adolescents (5, 8).

**Table 1:** Physical activity guidelines for children and adolescents in the selected countries.

Countries (Organisation)	First edition time	New version time	Name of the guidelines	Developers
Australia	2017	2019	Australian 24-h movement guidelines for children and young people (5–17 years)	Australian Government Department of Health Australian Council for Health^*^
Canada	2011	2016	Canadian 24-Hour Movement Guidelines for Children and Youth	Canadian Society for Exercise Physiology (CSEP); The Conference Board of Canada; Public Health Agency of Canada (PHAC); ParticipACTION^*^
China	2018	/	Chinese Physical Activity Guidelines for Children and Youth	National Children’s Medical Centre, Shanghai Children’s Medical Centre affiliated with Shanghai Jiao Tong University School of Medicine, Shanghai Institute of Physical Education
United Kingdom	2011	2019	UK Chief Medical Officers’ Physical Activity Guidelines	UK Government Department of Health of Social Care
United States	2008	2018	Physical Activity Guidelines for Americans 2nd edition	HHS, CDC, National Institutes of Health (NIH) Office of Disease Prevention and Health Promotion American College of Sports Medicine (ACSM)^*^
WHO	2010	2020	WHO 2020 Guidelines on physical activity and sedentary behaviour	WHO International Society for Physical Activity and Health (ISPAH)^*^

*Indicates a non-governmental organisation (NGO).

### 3.2 Guidelines recommended duration for physical activity

For a long time, scientific research focused on maintaining an extended, sustained state of moderate to vigorous physical activity (MVPA), such as the exercise behaviours of running, hiking and swimming (22). It was in the 1990s that the focus shifted to performing physical activity for no less than 10 min at a time throughout the day (9). The *2008 Physical Activity Guidelines for Americans* stated that any physical activity of more than 10 min daily could positively impact health (23). A large body of research also supports the idea that physical activity of 10 min or more per session can improve health (5, 24, 25). As research progresses, recent studies have found that MVPA of more than 1 min can contribute to health and that health status is related to the cumulative amount of physical activity (4, 9, 18). The latest evidence is consistent with the current recommendations in the new edition of the guidelines that engagement of MVPA for any duration contributes to health promotion.

Although there is considerable evidence of a positive association between physical activity and health status, research on the dose effects of physical activity in children and adolescents is limited compared to the studies in adults (5), i.e., further evidence is needed on exactly the minimum daily time that children and adolescents need to engage in physical activity to achieve optimal health benefits. Even so, it is generally accepted that a cumulative 60 min of MVPA per day in children and adolescents positively affects health status (9, 26). More than 60 min of MVPA can also provide additional health benefits (27). Therefore, 60 min per day is commonly recommended in national guidelines as the standard for time delineation. Previously, all the guidelines’ recommendations from the six countries and organisations, regarding the duration of physical activity for children and adolescents, were based on a “minimum” of 60 min per day. However, in 2019, researchers pointed out that the existing evidence did not support the minimum threshold of 60 min of MVPA per day as a recommendation (5, 27). Recent scientific evidence suggests that cumulative activity during the week is equally effective in promoting health (4). This finding provides help for children and adolescents who find it challenging to engage in at least 60 min of MVPA every day due to time constraints or barriers to physical activity. In light of these findings, the latest physical activity guidelines from the WHO and the United Kingdom recommend that children and adolescents strive to achieve an average of 60 min of MVPA per day over a week. This updated recommendation reflects a shift away from emphasising daily MVPA towards an emphasis on accumulated activity over time, which may better accommodate individual circumstances and preferences.

### 3.3 Guidelines recommended intensity for physical activity

Physical activity intensity refers to the amount of effort the body requires to perform a physical activity or exercise (27). Current guidelines from WHO and United States classify physical activity intensity into three categories: Low-intensity physical activity, Moderate-intensity physical activity, and Vigorous-intensity physical activity (4, 27). The need for children and adolescents to engage in MVPA daily has become a core guideline recommendation. The health benefits of MVPA are well-established in the academic community (28). Still, studies have shown that the higher the intensity of physical activity for the same duration, the greater the health benefits (4, 18). It suggests that regular, high-intensity physical activity is even more beneficial for cardiovascular health than other types of physical activity or a sedentary lifestyle (18, 29). A recent study on nearly 30,000 adolescents showed that physical activity intensity was a significant determinant of change in cardiometabolic risk factors. High-intensity physical activity significantly improves cardiovascular health in adolescents (30). Other studies have reported clinically significant effects on cardiovascular health, body weight and insulin resistance with High-Intensity Interval Training (HIIT) over short periods, supplemented by rest or recovery periods (18, 31), providing scientific evidence for improved cardiorespiratory health in children and adolescents who engaged in regular high-intensity exercise. These guidelines all recommend three additional sessions of vigorous-intensity physical activity (VPA) during the week, which was what the current guidelines differed from the previous ones (4, 9, 10, 15, 18). In addition to recommending MVPA and VPA, Canada and Australia considered the impact of light physical activity (LPA) on health status as equally important, supported by research evidence (12, 15). The most significant advantage of LPA is that it is straightforward to accomplish daily, even for unnoticed physical activities that can reach light intensity, such as leisurely walking or playing handball (15). The Canadian and Australian guidelines have included LPA in their daily activity guideline recommendations (12, 15), suggesting that several hours of LPA time are required daily (Table 2).

**Table 2:** Recommended physical activity types, intensities, durations and frequencies for children and adolescents by WHO and the selected countries.

The name of the guidelines	Age	Intensity			Type		
		Light	Moderate	Vigorous	Aerobic	Muscle	Bone
WHO 2020 Guidelines on Physical Activity and Sedentary Behaviour	5–17		MVPA averaging 60 min or more per day during the week 3 days per week VPA		MVPA is based on aerobic exercise, as well as muscle strengthening and bone strengthening activities		
UK Chief Medical Officers’ Physical Activity Guidelines	5–18		MVPA averaging 60 min or more per day during the week		Perform a variety of types and intensities of physical activity to enhance motor skills, muscle health and bone strength activities.		
Australian 24-h Movement Guidelines for Children and Young People (5–17 years)	5–17	Several hours per day	At least 60 min or more of MVPA per day, 3 days per week VPA		Daily activity includes a variety of aerobic exercises. Muscle and bone strengthening activities at least 3 days a week.		
Canadian 24-Hour Movement Guidelines for Children and Youth	5–17	Several hours per day	At least 60 min or more of MVPA per day, 3 days per week VPA		Daily activities include a variety of aerobic exercises. Muscle and bone strengthening activities at least 3 days a week.		
Physical Activity Guidelines for Americans (2nd edition)	6–17		At least 60 min or more of MVPA per day, 3 days per week VPA		MVPA is based on aerobic exercise, as well as muscle strengthening and bone strengthening activities		
Chinese Physical Activity Guidelines for Children and Youth	6–17		At least 60 min of cumulative MVPA per day		Daily MVPA with a focus on aerobic activity and 3 days a week of physical activity aimed at strengthening muscles and bones.		

MVPA, moderate to vigorous physical activity; VPA, vigorous physical activity.

### 3.4 Guidelines recommended frequency for physical activity

Frequency of physical activity refers to the number of times per week or per day an individual engages in physical activity (32). It is one of the key components of physical activity guidelines, along with duration, intensity, and type of activity. The frequency of physical activity is important because it affects the overall volume of physical activity that an individual accumulates over time. The frequency of physical activity recommendations for children and young people varies slightly among different guidelines. While 3/5 of the physical activity guidelines in this study recommend that children and adolescents engage in at least 60 min of MVPA per day (9, 10, 15), the WHO’s and UK’s guidelines state that MVPA can be accumulated in shorter bouts throughout the day and can be performed on most days of the week (5, 18). The latest scientific evidence suggests that while MVPA is recommended for children and adolescents for most days of the week, there is no evidence that one must engage in 60 min of MVPA every day to achieve health benefits. Research has shown that even small amounts of physical activity can have positive health effects, and that accumulating activity over the course of the week can be as effective as daily activity (4, 18). Additionally, some individuals may find it difficult to engage in MVPA daily due to time constraints or other barriers to physical activity. The WHO, United States, Australia, and Canada guidelines all recommend that children and adolescents engage in muscle-strengthening and bone-strengthening activities three times per week. Additionally, the guidelines from Australia and Canada specifically mention engaging in several hours of light-intensity physical activity each day.

### 3.5 Guidelines recommended types for physical activity

Aerobic exercise, muscle strengthening exercises and skeletal strengthening training are very beneficial to the health of children and adolescents (9, 18). The most important feature of the current guidelines for each country is the clarification of the types of physical activity, with the recommendation that all children and adolescents should focus on “aerobic exercise” and the addition of “muscle strengthening exercises” and “skeletal strengthening training” (Table 3). Aerobic capacity, muscle strength and bone strength are the basis of physical function and play an irreplaceable role in maintaining physical health. Improving aerobic capacity, muscular strength and skeletal strength will continue to benefit individuals throughout life (18).

**Table 3:** Examples of recommended physical activity programmes for children and young people in the guidelines from WHO and the selected countries.

Type of physical activity	Children	Adolescents
LPA	– Walking to school, walking a dog, going to park with friends, helping with household chores, playing handball	– Walking to school, walking the dog, going to the park with friends, helping with household chores, playing handball
MVPA*	– Brisk walking – Bicycling – Active recreational activities such as hiking, riding a small motorbike without an engine, swimming – Playing catch and throwing games such as baseball and softball – Soccer and basketball	– Brisk walking – Bicycling – Active recreational activities such as kayaking, hiking, swimming – Playing catch and throwing games such as baseball and softball – Household and yard work such as sweeping the floor or pushing the lawn mower – Some games that involve continuous action – Soccer and basketball
VPA*	– Running – Bike riding – Active games including running and chasing – Jumping rope – Cross-country skiing – Football, basketball, swimming, tennis and other sports – Martial arts – Fierce dancing	– Running – Bike riding – Active games involving running and chasing – Jumping rope – Cross-country skiing – Football, basketball, swimming, tennis and other sports – Martial arts – Fierce dancing
Muscle strengthening	– Games like tug-of-war – Resistance exercises using resistance bands – Rope climbing or tree climbing – Climbing on playground equipment – Yoga – Push-ups – Single bar – Sit-ups	– Games like tug-of-war – Resistance exercises using resistance bands, weight machines – Yoga – Push-ups – Single bar – Sit-ups
Bone strengthening	– Bouncing and bouncing – Jumping rope – Running – Sports that require jumping or rapid changes of direction	– Jumping rope – Running – Sports that require jumping or rapid changes of direction

*Some sports, such as cycling or swimming, can be either moderate or vigorous, depending on the level of effort put in World Health Organization (5).

#### 3.5.1 Aerobic exercise

For children and adolescents, aerobic exercise refers to rhythmic movements of large muscle groups performed continuously for a certain period of time. Activities such as running, jumping, skipping, swimming, dancing, and cycling are all considered aerobic exercise (4). Aerobic exercise can enhance cardiorespiratory fitness, but since children often engage in physical activity for short periods of time, strictly speaking, it may not meet the criteria for aerobic exercise. However, in order to provide precise terminology in the guidelines, even if children simply perform these activities, they are still referred to as aerobic exercise in the guidelines (9). The recommendations for aerobic exercise remain consistent across the countries, i.e., the majority of MVPA performed daily should be aerobic, and there should also be at least three sessions of high-intensity aerobic exercise per week.

#### 3.5.2 Muscle-strengthening exercises

Muscle-strengthening exercises represent activities in which muscle groups do more work than usual and can be referred to as ‘overload’ exercises (9). Muscle-strengthening exercises for children and young people can be unstructured activities such as tree climbing, tug-of-war, and using sports equipment in the playground. Muscle-strengthening exercises can be performed through organised activities such as weightlifting or resistance bands (27). Muscle-strengthening exercises can be done as part of daily physical activity, and children and adolescents should engage in muscle-strengthening physical activity at least 3 days a week (4, 9, 15).

#### 3.5.3 Skeletal strengthening exercises

Skeletal strengthening physical activity can produce stimulation of the bones, thereby promoting growth and strength. This stimulation is usually due to the reaction force of contact with the ground (18). Running, jumping rope, basketball and tennis are all bone-strengthening activities. Bone strengthening activities can combine aerobic and muscle strengthening, such as the various physical activities listed above (27). Bone-strengthening exercises can be done as part of 60 min or more of physical activity per day, and children and adolescents should do at least 3 days per week of bone strengthening (5).

### 3.6 How to assess intensity of physical activities?

It often needs to be clarified for parents and teachers for how to effectively and efficiently assess intensity when children and adolescents are engaged in physical activities. The physical activity guidelines suggest that intensity can be assessed in terms of relative and absolute terms (27).

#### 3.6.1 Relative intensity

Relative intensity is defined based on the individual’s physical condition. It indicates the level of effort exerted by the individual to complete the physical activity or the level of physical exertion felt during the physical activity (5). Relative intensity can be expressed as a percentage of a person’s maximum heart rate, heart rate reserve or percentage aerobic capacity reserve (9). For example, light physical activity is defined as activity that is below 50% of the maximum heart rate, and moderate-intensity physical activity has a range of maximum heart rate percentages between 63 and 76%. Physical activity above 76% of the maximum heart rate percentage is high-intensity physical activity (25). Relative intensity can be determined by assessing the perceived difficulty experienced by an individual during exercise, often using a scale ranging from 0 to 10, and described in relation to subjective exercise sensations. The Rating of Perceived Exertion (RPE) scale is commonly employed for this purpose (9). An intensity of 0 represents a resting state of inactivity, while an intensity of 10 represents intense physical activity performed to the best of one’s ability (9). In this scale, moderate intensity starts from level 5, and high-intensity physical activity starts from level 7 (Table 4). The need for children and adolescents to engage in daily aerobic activity to improve cardiorespiratory fitness is consistently recommended in the above-mentioned national guidelines. Relative intensity during exercise is a significant determinant of health outcomes. Therefore, it is feasible to measure children and adolescents in terms of relative intensity. When using relative intensity, people pay attention to how physical activity affects a child’s heart rate and breathing. For example, a person’s ability to speak during exercise can be used to assess the intensity of physical activity. Moderate-intensity activity allows a person to talk, but not to sing. In contrast, vigorous-intensity activity only allows them to say a few words before pausing for a breath (9). However, there are limitations when using relative intensity to measure physical activity in children and adolescents, as it is not guaranteed that the state of their activity can be observed at all times. In this case, absolute intensity can be used to determine whether the activity performed by the child follows the guidelines. It should be noted that the relative intensity as discussed above generally refers to aerobic type of exercise in the guidelines, however no specific methods were described for the muscle strengthening and bone strengthening activities in respect of their relative intensities.

**Table 4:** Criteria for discriminating physical activity intensity in children and adolescents.

Individual’s personal capacity	Intensity	Absolute Intensity	Subjective feeling	Max heart rate %
0	At rest	1 MET	Resting state	/
1	Sedentary behaviours	1–1.5 METs	Sedentary status	
2	Light intensity	1.6–2.9 METs	Extremely relaxing	<50
3			Very relaxing	
4			Relaxing	~63
5	Moderate intensity	3.0–5.9 METs	Breathing faster	~76
6			Shortness of breath	
7	Vigorous intensity	6.0 METs and above	Strenuous	~93
8			Very strenuous	≥94
9			Extremely strenuous	
10			Exhausted	100

METs, Metabolic Equivalents. Adapted from US Department of Health and Human Services (9).

#### 3.6.2 Absolute intensity

Absolute intensity is determined by the speed at which physical activity is completed without direct physiological assessments, such as cardiorespiratory fitness (33). The physiological effect of physical activity is energy expenditure. The measure of absolute intensity in physical activity is based on the rate of work being done and does not account for an individual’s physiological capacity. In aerobic exercises, the absolute intensity is commonly quantified by the rate of energy expenditure, expressed in metrics such as millilitres of oxygen consumption per kilogramme of body weight per minute, kilocalories per minute, or METs. For muscle-strengthening activities, the intensity is often indicated by the weight lifted or moved (9). When absolute intensity measures are used, light physical activity is generally considered in the guidelines to be non-sedentary waking behaviour with METs less than 3.0, for example, walking at a slow or leisurely pace (3 km/h or less), walking to school, helping with household chores, etc. Moderate intensity physical activity is defined as physical activity at 3.0 METs to 5.9 METs, e.g., walking briskly or purposefully (4–6 km/h), cycling, etc. High-intensity physical activity is defined as physical activity at 6.0 METs and above. Examples include brisk walking (7 km/h), running, rock climbing, cross-country skiing, etc. (Table 4).

### 3.7 Possible risks associated with physical activity

Physical activity can promote physical fitness in children and adolescents, but inevitably there are risks associated with the activity, commonly in the form of muscle or bone damage (5). The WHO and four other countries’ guidelines contain recommendations on the risks of physical activity. Activity risk factors are often associated with inadequate pre-exercise preparation activities, overly high emotions, substandard playing fields, and weather factors (34). However, this is lacking in the Chinese guidelines. The levels of physical activity recommended in the guidelines (dose effect) have not been found to pose a risk of injury (5), but children and adolescents should start by doing low-intensity physical activity when participating in activities and then gradually increase the frequency, intensity and duration, especially for children and adolescents who do not exercise regularly (9, 18), and should slowly and gradually increase the intensity of activity in a way that they enjoy. Gradually increasing the intensity, duration and frequency of exercise will help reduce the risk of injury. Eventually, children and adolescents should maintain daily participation in MVPA to meet the guideline recommendations and be more active if possible. Research indicates that exercising for more than 60 min a day can offer additional health benefits for children and adolescents (27). The United Kingdom guidelines state that the risk of adverse events from physical activity is relatively low and that the health benefits of physical activity far outweigh the risks (18). Canadian researchers have found in several studies that health risks (exercise accidents or injuries) increase slightly with increasing frequency and intensity of physical activity, but usually without severe consequences and that the health benefits of exercise far outweigh the risks posed (10). The health benefits of physical activity are clear, so physical activity promotion and accident prevention should be viewed in conjunction with each other rather than as opposing goals. Although there are potential risks associated with physical activity, the health benefits gained by following existing physical activity guidelines will far outweigh the potential risks.

## 4 Considerations in the future development of the physical activity guidelines for children and adolescents

There is considerable evidence indicating that the physical activity guidelines for children and adolescents are effective in promoting physical activity and reducing sedentary behaviour (4, 5, 25). The selected countries and organisations in this study, including Australia, Canada, China, United Kingdom, United States, and WHO, have conducted studies on physical activity in children and adolescents and developed corresponding guidelines systematically. This process includes a comprehensive review of scientific literature and consultation with relevant stakeholders. The development of these guidelines aims to promote physical activity, reduce sedentary behaviour among children and adolescents, and ensure that their physical activity levels meet recommended minimum standards for health. These guidelines are of great value and significance for improving physical activity levels in children and adolescents. The comparison reveals that there are both differences and commonalities in the guidelines of each country. Government agencies and NGOs in each country are the leading developers of the current guidelines, and the collective participation of government agencies and NGOs in the development of physical activity guidelines for children and adolescents can jointly promote the physical activity level of children and adolescents, and then improve the physical health of children and adolescents. The guidelines of Australia and Canada put forward the concept of LPA for several hours per day, and at the same time, the United States, Australia and Canada put forward the concept of 3 days a week VPA and emphasised the important role of VPA in health. The guidelines of WHO, the United States, the United Kingdom, Canada, and Australia were developed earlier and based on accumulated scientific research evidence, while the China’s guidelines were developed based on the contents of WHO, United States, United Kingdom, Canada and other countries’ guidelines prior to 2016 (21).

It is essential to base the physical activity guidelines for Chinese children and adolescents on evidence gathered within China. This approach acknowledges the unique cultural, lifestyle, and biological characteristics of this demographic, differing significantly from other populations. Customising these guidelines to align with China’s distinct environmental, socioeconomic, and health realities enhances their applicability and effectiveness. Relying on data specific to the Chinese context ensures the development of culturally relevant and accurate recommendations, thus promoting better health outcomes and facilitating more efficient policy implementation (8). This is because several factors may influence physical activity patterns among Chinese children and adolescents, which may differ from those in other countries. One factor is cultural and societal norms. Chinese culture values academic achievement, which may lead to a focus on sedentary activities such as school academic work, homework, additional tutorials after school, and screen time, rather than physical activity (35). Additionally, there may be cultural differences in attitudes towards physical activity, with some studies suggesting that Chinese children and adolescents may perceive physical activity as more difficult or less enjoyable compared to their Western counterparts (36).

Another factor is the built environment. Urbanisation and rapid economic development in China have led to changes in the built environment, which may impact physical activity patterns among children and adolescents. For example, increased motor vehicle use and reduced access to safe and accessible green spaces may limit opportunities for outdoor physical activity (37).

Furthermore, there may be differences in the prevalence and types of physical activity among Chinese children and adolescents compared to other populations. For example, some studies have suggested that Chinese children and adolescents may engage in less MVPA and more sedentary behaviour than their Western counterparts (38).

Overall, these factors suggest that physical activity guidelines for children and adolescents in China should be based on a different set of evidence collected from Chinese populations. Tailored guidelines that account for cultural and societal factors, as well as the built environment and prevalence of physical activity among Chinese children and adolescents, may be more effective in promoting physical activity and improving health outcomes in this population.

Although evidence suggests that physical activity guidelines for children and adolescents in China should be based on a set of evidence collected from Chinese populations, research data on physical activity in this population is limited. Therefore, it is important to refer to Western developed countries’ physical activity guidelines while developing tailored guidelines for Chinese children and adolescents. However, the current guidelines in China urgently need to be updated in line with the latest scientific evidence. Therefore, based on the findings of this study several recommendations for future revisions of the Chinese guidelines are presented below.

### 4.1 To update the recommended duration

The physical activity guidelines for children and adolescents in China currently recommend a ‘minimum’ of 60 min of MVPA per day. The guidelines are based on recommendations from the WHO, the United States, United Kingdom, Canada and other countries up to 2016. However, with the constant updating of scientific evidence in international guidelines, it is now accepted in the academic community that an “average” of 60 min of MVPA per day throughout the week is recommended. The updated guidelines have de-emphasised the previous focus on daily MVPA, and instead placed a greater emphasis on accumulating physical activity over longer periods of time. This approach is intended to better accommodate the unique circumstances and preferences of individuals.

### 4.2 To update the recommended intensity

Adolescents should not only engage in moderate-intensity physical activities. VPA is also very important and plays a crucial role in promoting the health and well-being of children and adolescents. Participating in VPA can bring more health benefits to adolescents, including cardiovascular health, bone and muscle health, and a reduced risk of chronic diseases. In addition, LPA also plays an important role in promoting the health of children and adolescents. The characteristic of LPA is that it is easy to accomplish in daily life, providing children and adolescents with opportunities for all-day activity, which can help improve overall physical health, increase energy expenditure, and reduce sedentary behaviour. Currently, the Chinese guidelines do not provide descriptions for VPA and LPA. It is recommended that any new Chinese guidelines emphasise the role of VPA and LPA, while also providing recommended levels of activity to promote the health and active lifestyle of children and adolescents.

### 4.3 To update the recommended frequency

The frequency of physical activity is crucial for achieving health benefits in children and adolescents. In terms of recommended frequency for muscle-strengthening and bone-strengthening exercises, the Chinese guidelines and those of other countries/organisations included in this study are consistent, with a minimum of three times per week. In terms of recommended frequency for MVPA, both China and countries such as the United States, Australia, and Canada recommend at least 60 min of MVPA per day. However, recent evidence suggests that it may be challenging for children and adolescents to engage in MVPA every day, and that the health effects of an average of 60 min MVPA per day over a week are similar to those of accumulated 60 min MVPA per day. In the future, the Chinese guidelines could consider referring to the recommendations from WHO and United Kingdom, which emphasise the accumulation of physical activity over a week. Additionally, the experience of guidelines from countries such as Australia and Canada could be drawn upon to recommend several hours of LPA per day, in order to reduce sedentary behaviour and improve overall physical activity levels in children and adolescents.

### 4.4 To update the recommended physical activity type

According to current evidence, it appears that fostering a broader and more varied repertoire of physical activity types in children and adolescents may confer notable benefits. This evidence supports the current guidelines for children and young people that support a range of different physical activities during the week. Children and adolescents should engage in a range of activities to improve their skills such as jumping, running and catching, as well as building the confidence to be active. Doing so has the potential to enhance motor skill development, bolster muscle health, and promote bone strength. Children and adolescents’ physical activity guidelines from Australia, Canada, United Kingdom, United States, and WHO currently emphasise the importance of engaging in a combination of aerobic, muscle-strengthening, and bone-strengthening exercises throughout the week, and provide recommendations regarding the number of different types of exercises to participate in each week. In future, guidelines in China could emphasise the significance of diverse exercise types and provide evidence-based recommendations regarding the frequency of participation per week.

### 4.5 To update recommendations for different age groups

Children and adolescents should meet the key guidelines by doing activities that are appropriate for their age. For example, children’s physical activities tend to be intermittent and unstructured plays, and as children grow into adolescents, their physical activity patterns will change. The Australia, Canada, the United Kingdom, the United States and WHO guidelines provide different recommendations for different age groups. However, the Chinese guidelines do not recommend targeted physical activities for children and youth at different ages. Future editions of the physical activity guidelines for children and adolescents in China could also tailor recommendations to different age groups, as younger children may have different physical activity needs compared to older adolescents.

### 4.6 To update methods for assessing PA intensity

The Chinese guidelines for assessing physical activity intensity in children and adolescents use a combination of pulse measurements and the RPE scale to determine relative intensity. On the other hand, the United States guidelines suggest that when adults supervise children, they may not be able to accurately measure a child’s pulse or breathing rate. However, they can still observe the type of activity a child is engaged in and determine whether it falls under moderate or vigorous intensity based on absolute energy expenditure. For instance, walking to school is an example of moderate-intensity activity, while running on the playground is considered vigorous-intensity activity. In future versions of the Chinese guidelines, methods for measuring the absolute intensity of physical activity in children and adolescents could be included to facilitate the assessment of their physical activity.

### 4.7 To update recommendations on how to identify and manage the risks

All the guidelines state that physical activity may carry the risk of injury, but appropriate protective measures can significantly reduce this risk. Conversely, physical inactivity is one of the most important risk factors for musculoskeletal injuries. Therefore, considering the balance between the health benefits and injury risks of physical activity in children and adolescents, it is still important to encourage them to engage in adequate levels of physical activity every day. Future Chinese guidelines could provide recommendations regarding the risks of exercise for children and adolescents at different levels of physical activity. Drawing from the experience of United States guidelines, those who do not meet the guidelines should be gradually introduced to MVPA in a manner that they prefer. This gradual increase in activity frequency and duration will help reduce the risk of injury. Children and adolescents who meet the guidelines should be encouraged to maintain their MVPA levels and increase their physical activity if possible. Those who exceed the guidelines should maintain their activity levels but may need to change the type of physical activity to avoid overtraining or injury.

Overall, future editions of the physical activity guidelines for children and adolescents in China could consider incorporating these recommendations to promote optimal physical activity and health among young people.

The limitation of this study is that the physical activity guidelines for children and adolescents in China primarily referenced the guidelines of the WHO, as well as those of the Australia, Canada, the United States and the United Kingdom, published in English, with additional references to the guidelines of two non-English-speaking countries, Japan and Germany. However, due to language limitations, only the physical activity guidelines from English-speaking countries were selected for this study. To further expand the boundaries of knowledge, it would be beneficial for future research to include new editions of the guide for non-English speaking countries. This would add to the existing guidelines and provide more comprehensive understanding and recommendations to a broader audience worldwide.

In addition, the role of the school environment in promoting physical activity among children and young people aged 6–17 years is essential to consider. While our analyses focused on specific guidelines, we found that these guidelines do not explicitly address how the broader school environments align with the FITT (frequency, intensity, time, and type) recommendations for physical activity. This observation suggests a need to broaden our perspective, considering the entirety of the school setting as a vital space for fostering physical activity. Prioritising an active school environment can significantly impact the health and well-being of children and adolescents. This approach involves not just structured physical education programmes but also the integration of physical activity into daily school routines and the creation of an environment that encourages and facilitates movement throughout the day. Given this context, further research is warranted to explore how the school environment, in its entirety, can be optimised to enhance the physical activity levels of children and adolescents. This would involve identifying strategies to make the entire school setting - including classrooms, outdoor spaces, and extracurricular activities - compatible with established physical activity guidelines. Such an integrated approach would ensure a more comprehensive and effective promotion of physical health and well-being in the school context.

## 5 Conclusion

This review presented a cross-sectional comparative study of the six latest sets of guidelines on physical activity for children and adolescents from Australia, Canada, China, the United Kingdom, the United States and WHO, analysing the history of the development of the guidelines; the policy context in which the guidelines were developed; and the main subjects of the guidelines. The core elements of the latest guidelines were identified and compared: duration, intensity, frequency, type of physical activity, assessment intensity of physical activity and risk prevention. There is an urgent need for the contents of the Chinese guidelines to be supported by the latest scientific evidence more specific to the cultural and socioeconomical environments of the population, and we therefore make the following recommendations for future revisions of the Chinese guidelines based on the findings of this study: (1) to update the recommended duration, intensity, frequency and type of physical activities that meet the requirements for Chinese children and adolescents; (2) to update methods for assessing physical activity intensity, and identifying and managing the risk factors in participating in physical activity; and (3) to incorporate analyses and reviews of school environment for effectively achieving the adequate levels of physical activity.

## Author contributions

DH: Conceptualization, Formal analysis, Funding acquisition, Writing – original draft. SZ: Conceptualization, Supervision, Writing – review & editing. ZC-M: Conceptualization, Supervision, Writing – review & editing. ZL: Methodology, Supervision, Writing – review & editing.
